# Mapping manifestations of parametric uncertainty in projected pelagic oxygen concentrations back to contemporary local model fidelity

**DOI:** 10.1038/s41598-021-00334-2

**Published:** 2021-10-22

**Authors:** U. Löptien, H. Dietze, R. Preuss, U. V. Toussaint

**Affiliations:** 1 Institute for Geosciences, University of Kiel, Ludewig-Meyn-Str. 10, 24118 Kiel, Germany; 2 IPP, Max-Planck-Institute for Plasma Physics, Garching, Germany

**Keywords:** Biogeochemistry, Climate sciences, Environmental sciences, Ocean sciences, Mathematics and computing

## Abstract

Pelagic biogeochemical models (BGCMs) have matured into generic components of Earth System Models. BGCMs mimic the effects of marine biota on oceanic nutrient, carbon and oxygen cycles. They rely on parameters that are adjusted to match observed conditions. Such parameters are key to determining the models’ responses to changing environmental conditions. However, many of these parameters are difficult to constrain and constitute a major source of uncertainty in BGCM projections. Here we use, for the first time, variance-based sensitivity analyses to map BGCM parameter uncertainties onto their respective local manifestation in model entities (such as oceanic oxygen concentrations) for both contemporary climate and climate projections. The mapping effectively relates local uncertainties of projections to the uncertainty of specific parameters. Further, it identifies contemporary benchmarking regions, where the uncertainties of specific parameters manifest themselves, thereby facilitating an effective parameter refinement and a reduction of the associated uncertainty. Our results demonstrate that the parameters that are linked to uncertainties in projections may differ from those parameters that facilitate model conformity with present-day observations. In summary, we present a practical approach to the general question of where present-day model fidelity may be indicative for reliable projections.

## Introduction

The utility of climate projections into an unacquainted future is strongly related to the knowledge about their associated uncertainties. Reliable estimates of uncertainties are, however, typically difficult to quantify and relate strongly to the choice of poorly known model parameters^1–3^.

Typically, these model parameters can not be observed directly and are, hence, difficult to determine. A generic approach is to compare the model output to observations and to choose a parameter set such that a desired model behavior is achieved. This desired model behavior is generally assessed with a *fidelity metric*, i.e. a weighted comparison between model output and specific observations. Naturally such a metric introduces a subjective element^4^ and relates closely to the question how to judge the quality of a specific model in general^5^. For an algorithm-based parameter optimization, the fidelity metric is generally represented by a single number, also known as *objective function* or *cost function*^6^. For practical reasons, a fidelity metric is based on data from past or contemporary eras^7–10^ and it is implicitly assumed that the metric is indicative of the reliability of future projections^3^. There is, however, no consensus on how to choose the underlying data. A notorious problem in climate science is that an assessment of the anthropogenic impact is limited to the past decades and we will not live to calculate substantiated statistics on the reliability of centennial climate projections. This puts it apart from, e.g., weather forecasting where decades of daily forecasts and subsequent ground truthing have quietly driven revolutionary success^11^. BGCM models, as generically implemented into the current generation of climate models, face the additional challenge that they are based on relatively recent developments; i.e. the underlying equations and concepts are still discussed controversially^12^. Limited data availability (only very few long-term time series are available and satellite observations refer to the sea surface only) and long wall-clock times (weeks to even months) to reach a quasi-equilibrium for each model formulation and parameter set, impede this process^10^. This puts these models even further apart from, e.g., the ocean and atmosphere modules of climate models that are built on first order principles such as Newton’s second law of Motion.

**Figure 1:**
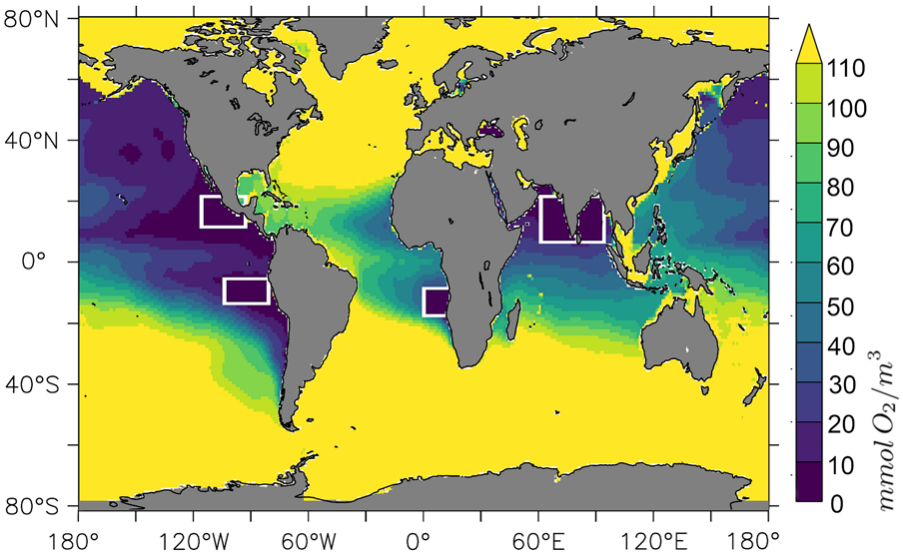
Illustration of regions where low, or even nil oxygen concentrations have been measured in the water column. The colours denote the lowest concentrations found in local water columns as archived by the World Ocean Atlas 2005^35^ in units ${\hbox {mmol}\,\hbox {O}_2\,\hbox {m}^{-3}}$. The white boxes mark the cores of notorious contemporary oxygen minimum zones (OMZs), as referred to in Table 1. This figure was created with PyFerret v7.5 (https://ferret.pmel.noaa.gov/Ferret/documentation/pyferret).

So why even bother with BGCM-based projections? The reason is that the pelagic oceanic nutrient, oxygen and carbon cycles were subject to pronounced changes during the past decades^13–15^ and we anticipate that these will continue as we move further into the Anthropocene. Among the associated pressing societal questions are the potential future developments of anthropogenic carbon storage in the oceans and oceanic deoxygenation - a term coined to describe the continuous decline of oxygen levels in the ocean which, in turn, may trigger a decline in fish yield^16^ and the release of the potent greenhouse gas nitrous oxide^17^. Such *Oxygen Minimum Zones (OMZs)* are particularly prominent in the tropical oceans (cf. Fig. 1). Thus, in response to urgent demands for information in order to facilitate mitigation and adaptation strategies, BGCMs are applied to project oceanic carbon and oxygen cycles - despite their limitations.

The current situation calls for strategies how to extent the existing (costly) observations to effectively reduce model uncertainties. In the present study, we illustrate a way forward to overcome some of the long-standing limitations associated with the notorious problem of assessing the reliability of BGCM-based projections. In order to quantify parameteric output uncertainties of these projections we employ variance-based methods^18,19^. More specifically, we use Sobol indices^20^ as representations of the sensitivities of a model with respect to the uncertainties of its input parameters and link the local manifestations of these parameter-induced uncertainties in now-casts of the simulated oceanic dissolved oxygen concentrations with those in future projections. We report on two methodological benefits. First, the method identifies regions of the World Ocean where parameter uncertainty induces particularly large uncertainties in future projections. This provides a spatial map of where the parameters induce large uncertainties and where the projected model results are relatively insensitive to the parameter uncertainties. Second, the method identifies regions where the current climate state is particularly sensitive to parameter changes. A variance decomposition quantifies the contribution of each of the (considered) parameters. In combination, the approach dissects the information of where contemporary observations may be used to constrain the model parameters and, with it, to confine the model spread (as induced by parameter uncertainty) among projections. These regions, where an increase in contemporary model fidelity maps on a reduced spread of projections are dubbed *benchmarking regions*. The approach proposed here allows to choose benchmarking regions depending on the specific region of interest in the projections (e.g. the projected oxygen minimum zones). Note that the respective favorable regions to constrain the model parameters under current climate conditions need not to be the same as the regions of particular interest in the projections, because the ocean circulation may map manifestations of parameter uncertainty from one region into another as time progresses.

This work is based on simulations with the UVic Earth System Model of intermediate complexity. The choice is motivated by the fact that (1) this model has been used to assess potential effects of geoengineering measures^21^ and, (2)—owing to its reduced complexity—it is computationally cheap which facilitates the piloting approach towards solving long-standing fundamental problems pursued here. We start from a frequently used reference configuration^22–24^, run the model under preindustrial conditions into quasi-equilibrium (by integrating a 2500 year-long spinup) and append a transient simulation by prescribing increasing atmospheric ${\mathrm{CO}}_2$ emissions until year 2100. The underlying emission scenario corresponds to RCP8.5^25^. In addition to the reference simulation, we integrate a corresponding ensemble, featuring 125 members. These ensemble members differ from the reference model version in that the following model parameters are perturbed within their uncertainty bounds (as derived from a literature survey^26–30^): (1) The sinking speed of detritus (*w*), (2) the vertical background diffusivity ($\kappa$) and (3) the maximum phytoplankton growth rate (*a*). This choice (out of $\approx$ 35 model parameters) is motivated by the parameters’ prominent roles in setting subsurface oxygen concentrations: Organic matter is produced by autotrophs in the sun-lit surface ocean controlled by the maximum phytoplankton growth rate (*a*). Cycling through the food web transforms part of this organic matter into detritus sinking at a velocity *w* to depth, where it is remineralized by heterotrophic bacteria. One major process balancing the respective oxygen consumption is vertical diffusion of oxygen from the sea surface (oxygenated by air-sea fluxes) to depth. The diffusive flux is typically calculated as the product of the vertical oxygen gradient and a diffusivity, compounding a constant background value ($\kappa$) with a dynamic parameterization^31^. Even though *w*, $\kappa$, and *a* are particularly influential, they are, at the same time, apparently impossible to constrain with ordinary contemporary data and common fidelity metrics, also because their interdependencies call for unrealistically high signal-to-noise ratios in observational data^3,30^.

The perturbed parameter ensemble is extended by Polynomial Chaos Expansion (PCE) to emulate additional model outputs and to, subsequently, apply the variance-based sensitivity analysis^32^. In the following we distinguish between the three time slices dubbed *preindustrial*, *contemporary*, and *projected* referring to year 1850, 2000 and 2100 respectively.

## Results

**Figure 2:**
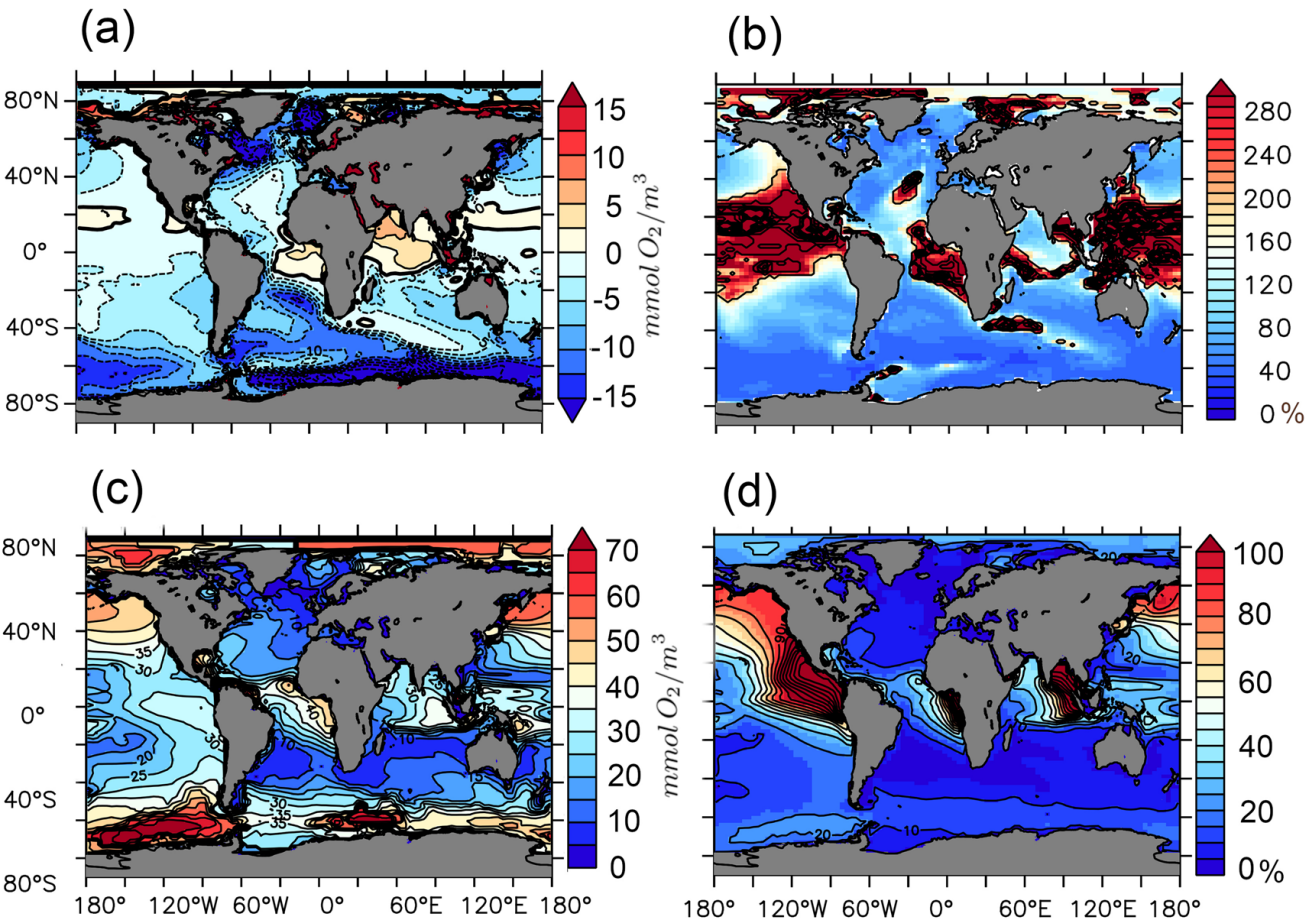
(**a**) Ensemble mean of simulated changes that accumulated from the preindustrial period to year 2100. Regions where the local minimum of oxygen concentrations is projected to decrease (increase) are denoted in blue (red). (**b**) Measure of the (parametric uncertainty-induced) spread of the projected changes (calculated as the standard deviation of the ensemble) normalized by the respective local amplitude of the ensemble mean changes. The unit of this noise-to-signal metric is %. Blue colours denote combinations of rather high anticipated changes with rather low parameter uncertainty. Red colours denote areas where anticipated changes are rather low and/or where the uncertainty as expressed by the ensemble spread is high. (**c**) Spread (calculated as standard deviation) among ensemble members reproducing the contemporary local minimum of oxygen concentrations in the water column. Red values denote regions where ensemble members differ widely. The unit is ${\hbox {mmol} \ \hbox {O}_2 \ \hbox {m}^{-3}}$. (**d**) Same as (**c**) but normalized by the contemporary ensemble mean of local minimum of oxygen concentrations. The unit is %. This figure was created with PyFerret v7.5 (https://ferret.pmel.noaa.gov/Ferret/documentation/pyferret).

Figure 2 summarizes our results in that it links local parameter-induced uncertainties in projected oxygenation with the (state-of-the-art^33^) model fidelity in reproducing the local and contemporary state of oceanic oxygenation. The first panel (Fig. 2a) shows the ensemble mean of projected changes in water column oxygenation, here defined as the minimum oxygen concentration found locally in the water column. One of the most prominent features of Fig. 2a is a substantial projected decrease in oxygenation in (and downstream of) all deep water formation sites, i.e., in the Greenland-Norwegian Sea, the Labrador Sea, the Mediterranean Sea, the Weddell Sea and the Ross Sea. At these sites global warming can map directly onto local ventilation by stabilizing the water column and by decreasing the (temperature-dependent) oxygen solubility. This in turn may explain the insensitivity towards the choice of the perturbed model parameters in our ensemble (shown as low noise-to-signal values in Fig. 2b, also referred to as the (local) coefficient of variation) at theses sites. Or, in other words, the choice of *w*, *a*, and $\kappa$ does hardly affect the projected changes of deoxygenation at deep water formation sites, relative to the strong signal that is associated with climate change. To this end, the ensemble of projections is rather insensitive to the parameter uncertainties—which is plausible since local ventilation is not directly affected by biotic processes (such as those controlled by *a* and *w*). Further, changes in the background diffusivity $\kappa$ apparently drives only minor differences at the deep water formation sites where deep convection events regularly exceed the background diffusivities by orders of magnitude (during deep water formation)—such that uncertainties in $\kappa$ do not retard the low noise-to-signal metric in the deep water formation sites shown in Fig. 2b.

Somewhat less prominent are projected changes in the tropics (Fig. 2a). These changes are small in absolute numbers and comprise typically values of the order of several $\hbox {mmol}\,\hbox {O}_{2}\,\hbox {m}^{-3}$ only. Even so, they can make substantial differences for the projected evolution of the oxygen minimum zones: e.g., decreasing the mean oxygen by 2 $\hbox {mmol}\,\hbox {O}_2\,\hbox {m}^{-3}$ yields a spatial extension of the suboxic volume by 30% (in the reference version of Uvic 2.9). Disconcertingly, the projections are especially uncertain in the tropics and OMZs, i.e., the noise-to-signal ratios in Fig. 2b are high, indicating that the uncertainty in model parameters maps onto a high spread amongst projections in these regions. Related questions are: Which processes are uncertain and which locations are best-suited for observations to constrain these processes (i.e., their associated model parameters)? The pragmatic approach to these questions implicitly assumes that the fidelity of reproducing local contemporary conditions is indicative of the reliability of its local projections. An essential precondition for this approach is that the existing model uncertainties are reflected in diverging contemporary local model responses.

By performing a variance-based sensitivity analysis^18,20^ we put the essential precondition to the test locally: Fig. 2c shows the respective extent to which ensemble members differ locally and contemporarily. It maps the local manifestation of differences among ensemble members. The main message here is that this local contemporary manifestation is not necessarily spatially correlated with the respective manifestations in future projections (i.e., Fig. 2b and d show different features). In the following, we put the information that is provided by the variance-based sensitivity analysis exemplarily to work and showcase the benefit of linking the local spread of projections with the local contemporary spread of the model ensemble (which is, essentially, a means of exploring the uncertainties of projections with local contemporary model fidelity).

**Figure 3:**
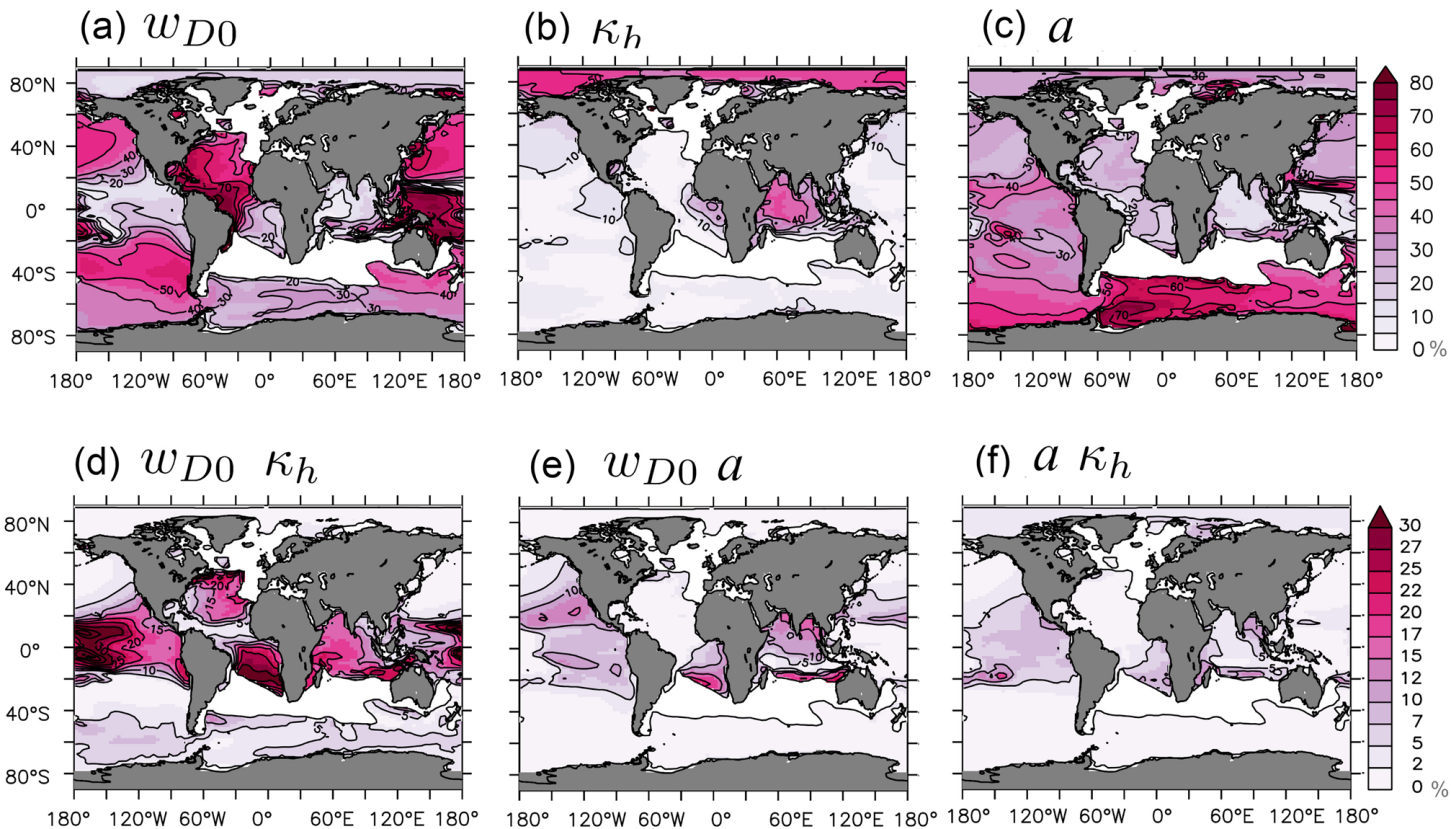
Variance-based sensitivity-analysis^20^ of the simulated minimal oxygen content in the water column in the year 2000. Panel (**a**–**c**) show the main effects for the considered model parameters (sinking speed of organic matter *w*, vertical background ocean mixing, $\kappa$, and maximum phytoplankton growth, *a*, resp.) and (**d**–**f**) illustrate the interactions. Regions where the overall variance is very small (standard deviation below 6% of the mean value) are masked out in white. This figure was created with PyFerret v7.5 (https://ferret.pmel.noaa.gov/Ferret/documentation/pyferret).

For the Atlantic OMZ, we find both contemporary and projected sensitivities being relatively high (Fig. 2b and d). Further, the uncertainty in projections is linked in roughly equal terms to the uncertainties of all three model parameters (Table 1) which also applies to contemporary ensemble spread (Fig. 3a and b). Also, sinking speed of organic matter, *w*, and vertical mixing, $\kappa$, show relatively strong interactions in simulated present climate. Well-suited locations for measurements to constrain the respective model parameters are indicated by a large spread in the perturbed parameter ensemble (Fig. 2d). Ideally, the spread could predominantly be attributed to a single parameter, because this would facilitate the parameter estimation substantially and avoid potential problems due to mutual dependencies^30^ (i.e. we propose to combine the Sobol indices with a preferably high ensemble spread while regions with very low variance are masked out in Fig. 3). For the mixing parameter $\kappa$ relatively high contemporary and exclusive manifestation is given in the Indian Ocean (Figs. 2d and 3). We conclude that the Indian Ocean is a contemporary benchmarking region for $\kappa$ and that proper use of observations at this site can reduce parameter uncertainties of projections of the Atlantic OMZ. Somewhat counter to intuition, constraining $\kappa$ (be it with data from the Indian Ocean or elsewhere) is rather irrelevant for projecting the Indian Ocean OMZs with our model. There, along with the North and South Pacific OMZs the phytoplankton growth parameter *a* is most influential (Table 1). Figure 3 suggests that for the simulated minimal oxygen concentrations in our model, the Southern Ocean is a well-suited contemporary benchmarking regions to constrain *a* with contemporary observational data. Or in other words—as *a* is the most influential parameter in projections of the OMZ in the Pacific and Indian Oceans, being able to simulate the oxygen concentrations in the Southern Ocean is a prerequisite in our model to more reliably project these OMZs.

In summary, we advocate the application of the method of Sobol to explore links between parameter uncertainties, local contemporary model-data misfits and local uncertainties of projections into our warming future. A straightforward application is the identification of contemporary benchmarking regions to constrain the model parameters. To project the OMZs in the Pacific and Indian Oceans more reliably, a good representation of the oxygen concentrations in the Southern Ocean is a necessary (although not sufficient) condition in our model framework. For the Atlantic OMZ we could not identify such a clear benchmarking region and all considered parameters are almost equally influential. As, however, a large model spread of the perturbed parameter ensemble occurs in the northern Pacific and the Atlantic Ocean, oxygen measurements in these regions are advised.

As a side aspect of the presented results, we report that the long-standing problem of a biased Indian Ocean OMZ, apparently endemic to the current generation of coupled ocean-circulation biogeochemical models^34^, is in our model related to the representation of vertical mixing processes rather than to unknowns in biogeochemical model parameters.

**Table 1:** Measure of the contribution of respective parameter uncertainties to the variance (spread) within  the ensemble of projected changes of the local oxygen minima.

Region	*w* (%)	$\kappa$ (%)	*a* (%)	*w* $\kappa$ (%)	*w* *a* (%)	$\kappa$ *a* (%)
Atlantic	26.37	27.28	22.50	5.312	11.21	7.021
Indian Ocean	26.82	14.00	34.82	1.176	15.40	2.745
Pacific-north	27.09	20.53	33.36	2.421	12.71	3.774
Pacific-south	12.20	4.016	74.37	0.610	6.037	2.085

The units are %, calculated using the method of Sobol^20^. The values refer to spatial averages of the regions defined in Figure 1.

## Discussion

We presented, for the first time, spatial maps linking contemporary benchmarking regions with the uncertainty of projections of pelagic biogeochemistry that is associated with uncertain parameter settings. Among the results is that, in our model framework, the contemporary Indian Ocean is a benchmarking region for vertical background mixing which needs to be constrained to project the Oxygen Minimum Zone in the Atlantic Ocean. In order to reduce the parametric uncertainties of forecasts for the Indian Ocean OMZ, however, phytoplankton growth needs to be constrained—preferably with observations in the Southern Ocean which provides for our model the best benchmarking region for this purpose.

Caveats apply. For one, state-of-the-art numerical representation of pelagic biogeochemistry are not rooted in first principles; they were developed for interpolation tasks and it is not clear whether it is admissible to employ them for projections because, e.g., they may fail to incorporate effects of biotic adaptation. Second, our approach addresses parametric uncertainty only. Unresolved processes and other sources of uncertainty are not included and the transferability to other emission scenarios must be investigated. Third, variance-based sensitivity analysis are computationally costly because the method necessitates large ensembles to make statistically meaningful inferences^36^. Here we were able to restrict the cost to feasible levels by using Polynomial Chaos Expansion (PCE)^37–39^. Also, neural network-based approaches are ermerging^40^.

## Methods

### Uncertainty quantification and Sobol indices

The presented study is based on a variance-based global sensitivity analysis using Sobol indices^18,20,41^ which is applied to each model grid point individually. Working within a probabilistic framework, we decompose the variance of a model output of interest into fractions that can be attributed to the poorly known model parameters (here sinking speed of detritus (*w*), the vertical background diffusivity ($\kappa$) and the maximum phytoplankton growth rate (*a*)). The method is based on the calculation of conditional expected values and provides percentages that are directly interpreted as measures of sensitivity. Such an approach to uncertainty quantification is attractive, because (1) the sensitivity is measured across the whole space of input parameters, (2) the method can deal with nonlinear responses and (3) it can measure the effect of interactions in non-additive systems^42^. Variance-based sensitivity analysis has, however, the disadvantage of being computationally particularly costly because it requires a multitude of model simulations under various parameter settings. To reduce the computational burden to a feasible level, we restrict ourselves to a non-intrusive reduced-order model approach within the framework of uncertainty quantification based on *Polynomial Chaos Expansion (PCE)*^37–39^. This approach limits the number of required model simulations substantially by using relatively few model simulations as collocation points for a spectral fit with Hermite basis functions^43^. In our study we consider Hermite polynomials up to third order because the contributions of higher orders become numerically insignificant (i.e., their relative values being lower than machine accuracy). We use 125 model simulations with input parameters being set by a Gaussian quadrature.

We assume the parameters to be normally distributed within a standard deviation of roughly 50% for *w* and $\kappa$ and 25% for *a*. These values are well within the range suggested by foregoing studies on parameter uncertainties^27,30,44,45^. Occasional negative and very small values are truncated because these lead to unrealistic model results. The mean values refer to the reference model version UVic 2.9^46^ which was tuned to match the climatological observations by the World Ocean Atlas^47^. Further details on the Earth System model and the parameter settings are provided in the supplement.

## Supplementary Information

Supplementary Information.

## Data Availability

The analysed model output is archived at https://zenodo.org/record/5537081#.YVW1t8ZCR0s. The data are distributed under the MIT Expat Licence.

## Code availability

All code is written in Fortran. The code for the sensitivity analysis may be used under the MIT Expat Lizenz. It is archived at https://zenodo.org/record/5537081#.YVW1t8ZCR0s under 10.5281/zenodo.5537081. The UVic ESCM model was developed by the University of Victoria and the current version was refined at GEOMAR Helmholtz Centre for Ocean Research Kiel^46^. The UVic ESCM is provided freely without restrictions other than the requirement that users not use it to participate in model intercomparison projects without prior permission (https://thredds.geomar.de/thredds/catalog/open_access/uvic_kiel_2016/catalog.html).
