# Supplementary material for: Mapping manifestations of parametric uncertainty in projected pelagic oxygen concentrations back to contemporary local model fidelity
Source: Sci Rep. 2021 Oct 22;11:20949. doi: 10.1038/s41598-021-00334-2 (PMC8536705; doi:10.1038/s41598-021-00334-2)
Supplement: Supplementary file 1 — Supplementary Information. [file 41598_2021_334_MOESM1_ESM.pdf]

Mapping manifestations of parametric uncertainty in projected pelagic oxygen concentrations back to contemporary local model fidelity - Supplement

U. Löptien[1]*, H. Dietze[1], R. Preuss[2], U. v. Toussaint[2]

[1]*Institute for Geosciences, University of Kiel, Ludewig-Meyn-Str. 10, 24118 Kiel, Germany*

[2]*IPP, Max-Planck-Institute for Plasma Physics, Garchingen, Germany*

* *Correspondence: ulrike.loeptien@ifg.uni-kiel.de*

1 The Reference Model

Our reference model (REF), UVic 2.9, is of intermediate complexity and has a horizontal resolution of 1.8° in latitude and 3.6° in longitude in all submodules. The model includes a single-level atmospheric energy-moisture balance model (with prescribed surface winds from the NCAR/NCEP monthly climatology), a dynamic-thermodynamic sea ice model, a simple land ice model and an active terrestrial vegetation component [1]. Further, the Earth System Model includes an ocean component which is based on a three-dimensional primitive-equation model [2]. The vertical discretisation of the ocean comprises 19 levels and increases gradually from $50\,m$ at the surface to $500\,m$ at depth. The vertical background mixing parameter, κ_h is constant - apart form the Southern Ocean (south of 40°S) where the background value is increased by a value of 1.0 $\mathrm{cm^2 s^{-1}}$. κ_h is poorly constrained in ocean models and there are indications that this parameter might add considerable uncertainties to simulated oxygen concentrations [3]. An anisotropic viscosity scheme [4] is

implemented to improve the equatorial circulation [5]. The ocean component is coupled to a marine pelagic ecosystem model [6]. It's prognostic variables are phytoplankton (Phyt), diazotrophic phytoplankton (PD), zooplankton (Z), detritus (D), nitrate (NO3), phosphate (PO4), dissolved oxygen (O_2), dissolved inorganic carbon (DIC), and alkalinity (ALK).

The temporal evolution of each these prognostic variables is given by a partial differential equation:

$$\frac{\partial C}{\partial t} = T + S. \tag{1}$$

C stands for the respective prognostic variable, T denotes the convergence (or divergence) of physical transports (i.e., the sum of advection, isopycnal and diapycnal diffusion) and S denotes the source minus sink terms (such as differences between growth and loss, air-sea fluxes, sinking). The model is described in detail in the respective model description papers [6,7]. Here, we present a choice of details which refer to the considered biogeochemical parameter changes (i.e. the sinking of detritus, w_{D0}, and the remineralization of detritus, μ_{D0} - see also Table S1).

In our model phytoplankton growth (J_D) depends on photosynthetically active radiation (PAR), nitrate (NO_3), phosphate (PO_4) and iron (Fe) (where the latter is parameterized by an iron mask rather than explicitly resolved). The specific formulation is as follows:

$$J_D = a\ J_{Fe}\ min(J_{OI}, J_{NO_3}, J_{PO_4}), \tag{2}$$

where a determines the maximum phytoplankton growth and J_{Fe} mimics iron limitation and temperature dependence:

$$J_{Fe} = \frac{Fe}{Fe + K^p_{Fe}} \cdot exp(T/T_b). \tag{3}$$

The parameter K^p_{Fe} is the so called *half saturation constant* for iron limitation and T_b the e-folding temperature of biological rates.

Light limitation is expressed as:

$$J_{OI} = \frac{\alpha\, PAR}{((\alpha\, PAR)^2 + (J_{Fe})^2)^{(1/2)}}, \tag{4}$$

where the parameter α determines the sensitivity toward the incoming photosynthetically active radiation (initial slope of the P-I curve).

Growth limitations due to lack of nitrate (NO_3) and phosphate (PO_4) are expressed by the so called *Michaelis-Menten (MM)-formulations*. The sensitivity is towards the availability of these nutrients is determined by the halft saturation constants K_N, resp. K_P (which are related via the Redfield ratio $R_{N:P}$=1/16; i.e., K_P=$R_{N:P}$ K_N):

$$J_{NO_3} = \frac{NO_3}{NO_3 + K_N} \tag{5}$$

and

$$J_{PO_4} = \frac{PO_4}{PO_4 + K_P}. \tag{6}$$

Phytoplankton blooms are terminated by zooplankton grazing once essential nutrients are depleted. Both, phytoplankton and zooplankton produce detritus which the sinks to depth. This sinking speed determines, in combination with the remineralization rate, the depth at which detritus is converted back into dissolved species (such as nitrate, phosphate, DIC) and thus ends its route

down to the sea-floor. The sinking speed of detritus, w_D, increases linearly with depth:

$$w_D = w_{D0} + m_W z,$$

where w_{D0} is the detritus sinking speed at the surface, m_W denotes depth dependent detritus sinking speed and z is the effective vertical coordinate (positive downward). In one of our configurations we change the value of the model parameter w_{D0}. Remineralization of detritus returns the nitrogen (N) and phosphorus (P) content of detritus back to NO_3 and PO_4, consumes oxygen and releases inorganic carbon - which then completes the cycle of nutrients.

Table S1: Model parameters, their reference values and their bounds for sensitivity analysis, considered in this study. The range is given by a standard deviation. We assume respective normal distributions for the parameter ranges which are centered around the reference values. Occasional negative and too small values are replaced by the respective lower thresholds (0.5, 0.06 and 0.08 for κ_h, w_{D0} and μ_{D0}, resp.), because lower values erode the model behaviour and are not meaningful

Param.	Description	Unit	Ref. Value	Standard deviation
κ_h	Vertical background diffusivity	$\mathrm{cm^2 s^{-1}}$	0.15	0.08
w_{D0}	Detritus sinking speed at the surface	$\mathrm{m\ day^{-1}}$	12.5	6.0
a	Maximum phytoplankton growth rate	$\mathrm{day^{-1}}$	0.6	0.15

Experiments All model versions start from the equilibrated state of what is dubbed *reference run* in [8] and are integrated for 2500 model years with constant, pre-industrial CO_2 emissions. This reference run refers to parameter values which have been subjectively tuned to fit observations summarized in the World Ocean Atlas [9]. Also other foregoing studies [3,10] showed a rather smooth model response towards the parameter changes and no extreme outliers were detected. The simulations which form the base of the variance-based sensitivity analysis start from these spunup simulations which are then continued for another 300 years with varying CO_2-emissions, covering the period 1850-2150. These experiments start with the historical state and are continued with the CO_2 emission scenario RCP 8.5 [11]. The present climate state refers to a 10 year average, covering the year 2000, while the projected changes refer to the simulated difference between the years 2100 and 1900.

2 Variance-based Sensitivity Analysis and Uncertainty Quantification

Based on our prior assumption about the parameter uncertainties being normally distributed we employ a spectral expansion to quantify the propagation of uncertainty through the model. To limit the number of required model simulations substantially we use a polynomial fit with Hermite polynomials as orthonormal basis functions between a discrete set of collocation points (consisting of a model output of interest) [12]. This approach is known as 'polynomial chaos expansion', but mathematically represents a 'spectral expansion'. Once successfully determined, the spectral representation is capable of quantifying the uncertainty for any point in the model output space or to serve as a surrogate model. This approach is non-intrusive, but approximate in the sense that the

mathematical infinite spectral expansion is confined to a finite sum. The emerging integrals in the calculation of the polynomial coefficients are evaluated by Gaussian quadrature which identifies the collocation points with those of the quadrature. Moreover, we assume (at least approximately) mutually independent normally distributed random variables.

To quantify the uncertainty of a model result R we seek the appropriate function $g(\xi)$, such that R will have the required distribution of the model response, $R = g(\xi)$. For all random variables with finite variance it is possible to at least find an infinite expansion:

$$g(\xi) = \sum_{k=0}^{\infty} a_k \psi_k(\xi) \approx \sum_{k=0}^{P} a_k \psi_k(\xi) \,. \tag{7}$$

We limit the infinite expansion to a polynomial of order P when the contributions of higher orders become (typically) numerically insignificant (in our example P=3). The coefficients are given by:

$$a_k = \frac{\langle g(\xi), \psi_k(\xi) \rangle}{\langle \psi_k(\xi), \psi_k(\xi) \rangle} \,, \qquad \text{with} \quad \langle g(\xi), \psi(\xi) \rangle = \int g(\xi) \psi(\xi) p(\xi) \mathrm{d}\xi \,. \tag{8}$$

We assume Gaussian character for the random variable, so the density $p(\xi)$ is distributed according to the normal (probability) distribution

$$p(\xi) = \frac{1}{\sqrt{2\pi}} \exp\left\{ -\frac{\xi^2}{2} \right\} \,. \tag{9}$$

The adjunctive set of orthonormal basis functions is given by the so-called *probabilist* Hermite

functions, which read up to third order as:

$$\begin{aligned}\psi_0(\xi) &= 1\,,\\ \psi_1(\xi) &= \xi\,,\\ \psi_2(\xi) &= \xi^2 - 1\,,\\ \psi_3(\xi) &= \xi^3 - 3\xi\,,\end{aligned} \tag{10}$$

With these definitions the normalization constants in Eq. (8) can be written as

$$\langle \psi_k, \psi_k \rangle \;=\; \int \psi_k(\xi)\psi_k(\xi)p(\xi)\mathrm{d}\xi \;=\; k! \quad . \tag{11}$$

Due to the Gaussian nature of the probability functions in the integrals above, it is beneficial to use Gauss-Hermite (GH) quadrature for the evaluation

$$\langle g(\xi), \psi(\xi) \rangle \overset{GH}{=} \sum_{l=0}^{L} g(\xi_l)\psi(\xi_l)w_l\,, \tag{12}$$

where the weights w_l and the abscissas ξ_l are for instance provided by Numerical Recipes [13]. Eventually, by exploiting the properties of the orthogonal Hermite polynomials the expectation value of the model outcome and its variance can be assigned to the spectral coefficients in Eq. (8)

$$\langle R \rangle = a_0\,, \qquad \mathrm{var}(R) \;=\; \langle R^2 \rangle - \langle R \rangle^2 \;=\; \sum_{k=1}^{P} a_k^2 k!\,. \tag{13}$$

In order to provide a measure for the influence of the uncertainty of input parameters on the above variance we employ Sobol coefficients [14] (i.e. a decomposition of the model output variance to the individual input parameters or their combinations). These are defined by

$$S_i = \frac{D_i}{\mathrm{var}(R)}\,, \qquad S_{ij} = \frac{D_{ij}}{\mathrm{var}(R)}\,, \qquad \dots \quad , \tag{14}$$

where the evaluation of the integrals

$$
\begin{aligned}
D_i &= \int g_i^2(\xi_i)\mathrm{d}\xi_i \quad , \\
D_{ij} &= \int\int g_{ij}^2(\xi_i,\xi_j)\mathrm{d}\xi_i\mathrm{d}\xi_j \quad , \\
... & \quad ,
\end{aligned}
\tag{15}
$$

results in combinations of the coefficients of Eq. (8) (the index of the function $g_{index}(...)$ relates to the specific variable(s) ξ_{index} which are omitted in the integral $g_{index} = \int ... \int g(\vec{\xi})\mathrm{d}\xi_{\{index\}}$). The higher the value of a Sobol coefficient with respect to the others is, the higher is the contribution of the respective model parameter to the ensemble spread (variance).

References

1. Weaver, A.J., Eby, M., Wiebe, E.C., Bitz, C. M. , Duffy, P.B. et al. The UVic Earth System Climate Model: Model description, climatology, and applications to past, present and future climates. *Atmosphere-Ocean* **39,** 361-428 (2001).

2. Pacanowski, R.C. MOM 2 documentation, user's guide and reference manual. GFDL *Ocean Group Tech. Rep* **3,** 232 (1995).

3. Löptien, U. & Dietze, H. Reciprocal bias compensation and ensuing uncertainties in model-based climate projections: pelagic biogeochemistry versus ocean mixing. Biogeosciences, **16,** 1865-1881 (2019).

4. Large, W. G., Danabasoglu, G., McWilliams, J. C., Gent, P. R. & Bryan, F. O. Equatorial circulation of a global ocean climate model with anisotropic horizontal viscosity, *J. Phys. Oceanogr.,* **31,** 518-536 (2001).

5. Somes, C. J., Schmittner, A., Galbraith, E. D., Lehmann, M. F., Altabet, M. A. et al. Simulating the global distribution of nitrogen isotopes in the ocean, *Global Biogeochem. Cycles* **24,** GB4019 (2010).

6. Keller, D.P., Oschlies, A. & Eby, M. A new marine ecosystem model for the University of Victoria Earth System Climate Model, *Geoscientific Model Dev.* **5,** 1195-1220 (2012).

7. Schmittner, A., Oschlies, A., Matthews, H. D. & Galbraith, E.D.: Future changes in climate, ocean circulation, ecosystems, and biogeochemical cycling simulated for a business-as-usual CO_2 emission scenario until year 4000 AD, *Global Biogeochemical Cycles*, **22,** GB1013 (2008).

8. Getzlaff, J. & Dietze, H. Effects of increased isopycnal diffusivity mimicking the unresolved equatorial intermediate current system in an earth system climate model. *Geophys. Research Letters* **40**, 2166-2170 (2013).

9. Garcia, H. E., Locarnini, R. A., Boyer, T. P. , Antonov, J. I. , Zweng, M.M. et al. *World Ocean Atlas 2009, Volume 4: Nutrients (Phosphate, Nitrate, Silicate)* (S. Levitus, Ed. NOAA Atlas NESDIS 71, U.S. Govern. Print. Office, Washington, D. C, 2009).

10. Löptien, U. & Dietze, H. Effects of parameter indeterminacy in pelagic biogeochemical modules of Earth System Models on projections into a warming future: The scale of the problem. *Global Biogeochemical Cycles* **31,** 1155-1172 (2017).

11. Riahi, K., Rao, S., Krey, V., Cho, C., Chirkov, V. et al. RCP 8.5 - A scenario **of** comparatively high greenhouse gas emissions. *Climatic Change* **109,** 33 (2011).

12. Preuss, R. & von Toussaint, U. *Investigations on bayesian uncertainty quantification with two examples*, (A. Giffin, K. Knuth (Eds.), Bayesian Inference and Maximum Entropy Methods in Science and Engineering, Vol. 1757, AIP Publishing, Melville, NY, 2016).

13. Press, W.H., Teukolsky, S.A., Vetterling, W.T., Flannery, B. P. *Numerical Recipes: The Art of Scientific Computing* (3rd Edition, Cambridge University Press, 2007).

14. Smith, R.C. *Uncertainty Quantification: Theory, Implementation, and Applications* (SIAM, Philadelphia, 2014).
